# Supplementary figures and images for: Cluster size distribution of cells disseminating from a primary tumor
Source: PLoS Comput Biol. 2021 Nov 10;17(11):e1009011. doi: 10.1371/journal.pcbi.1009011 (PMC8608333; doi:10.1371/journal.pcbi.1009011)

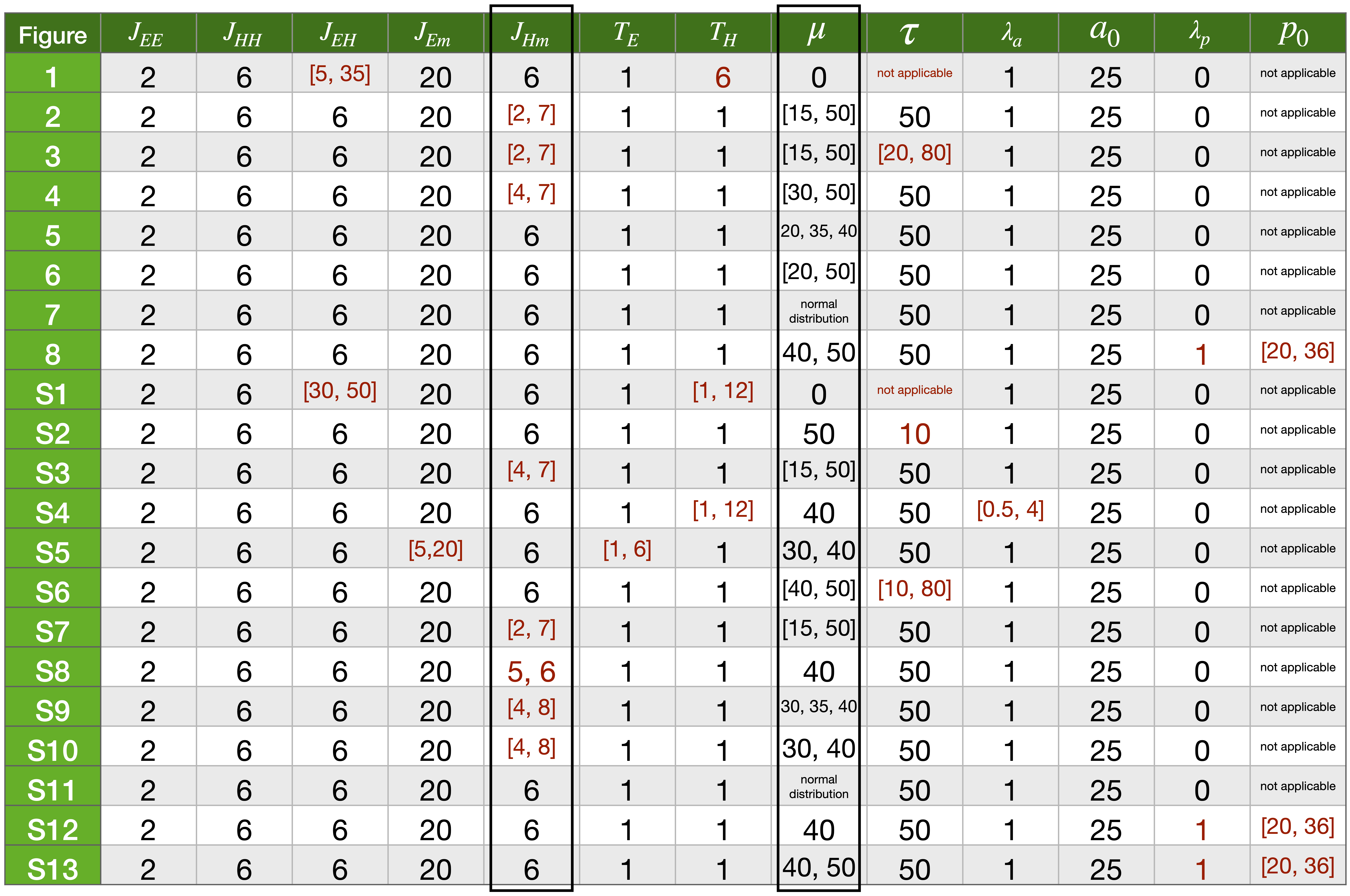

Supplement: S1 Table — We use surface tensions (γ) instead of contact energies (J), defined as γEm = JEm − JEE/2, γHm = Γ = JHm − JHH/2. The uncommon ones which are different from the standard parameters used in most of the simulations are colored in red. We mainly focus on the two parameters JHm (or in other words γHm = Γ) and μ related to H cells in this article. (TIFF) [file pcbi.1009011.s003.tiff]

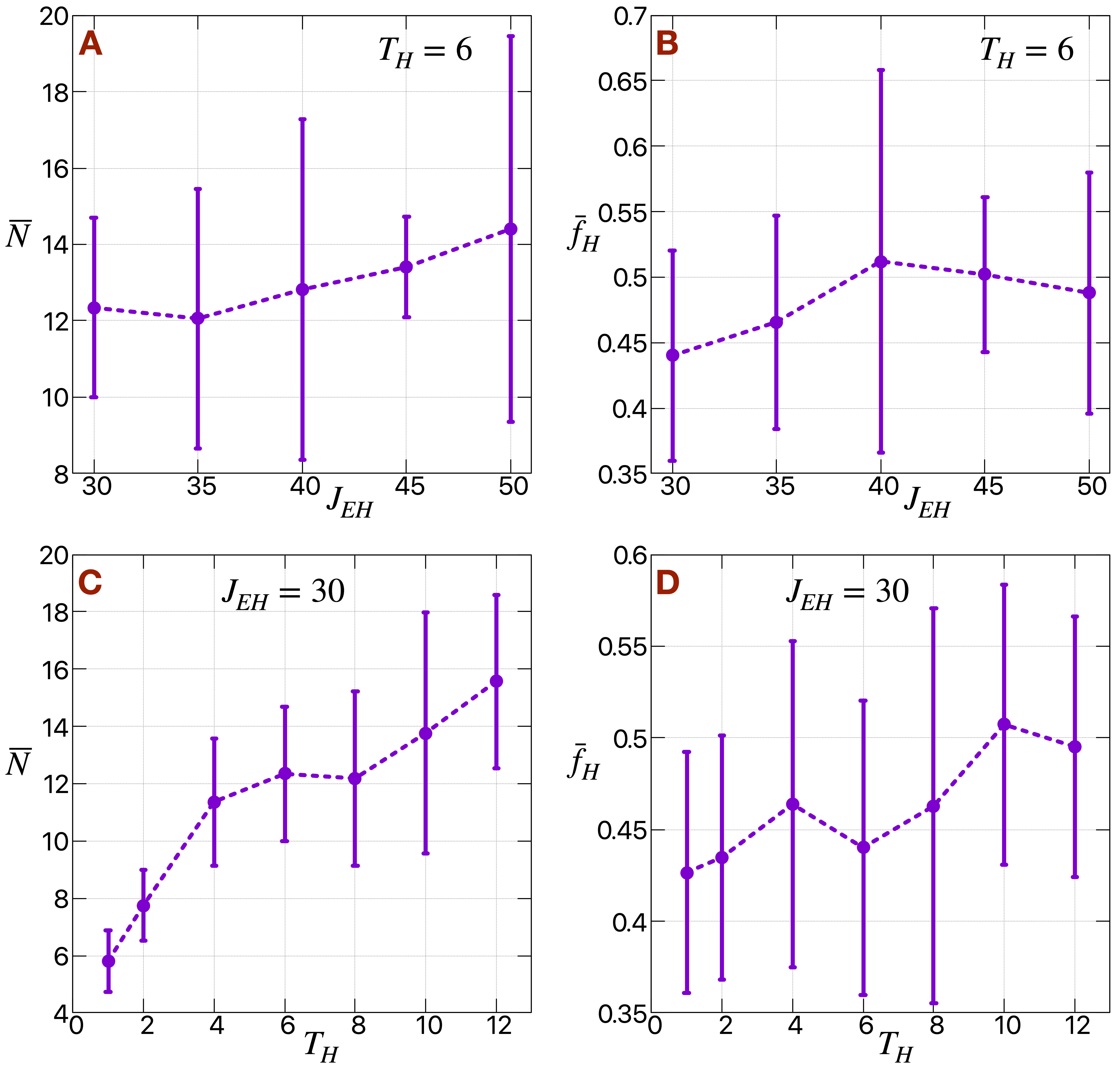

Supplement: S1 Fig — The (A) mean cluster size (N¯) of H cells and (B) fraction of H cells (f¯H) released in the medium as a function of JEH at TH = 6. The (C) mean cluster size (N¯) of H cells and (D) fraction of H cells (f¯H) released in the medium as a function of TH at JEH = 30. We use standard deviations of the data as error bars. (TIFF) [file pcbi.1009011.s004.tiff]

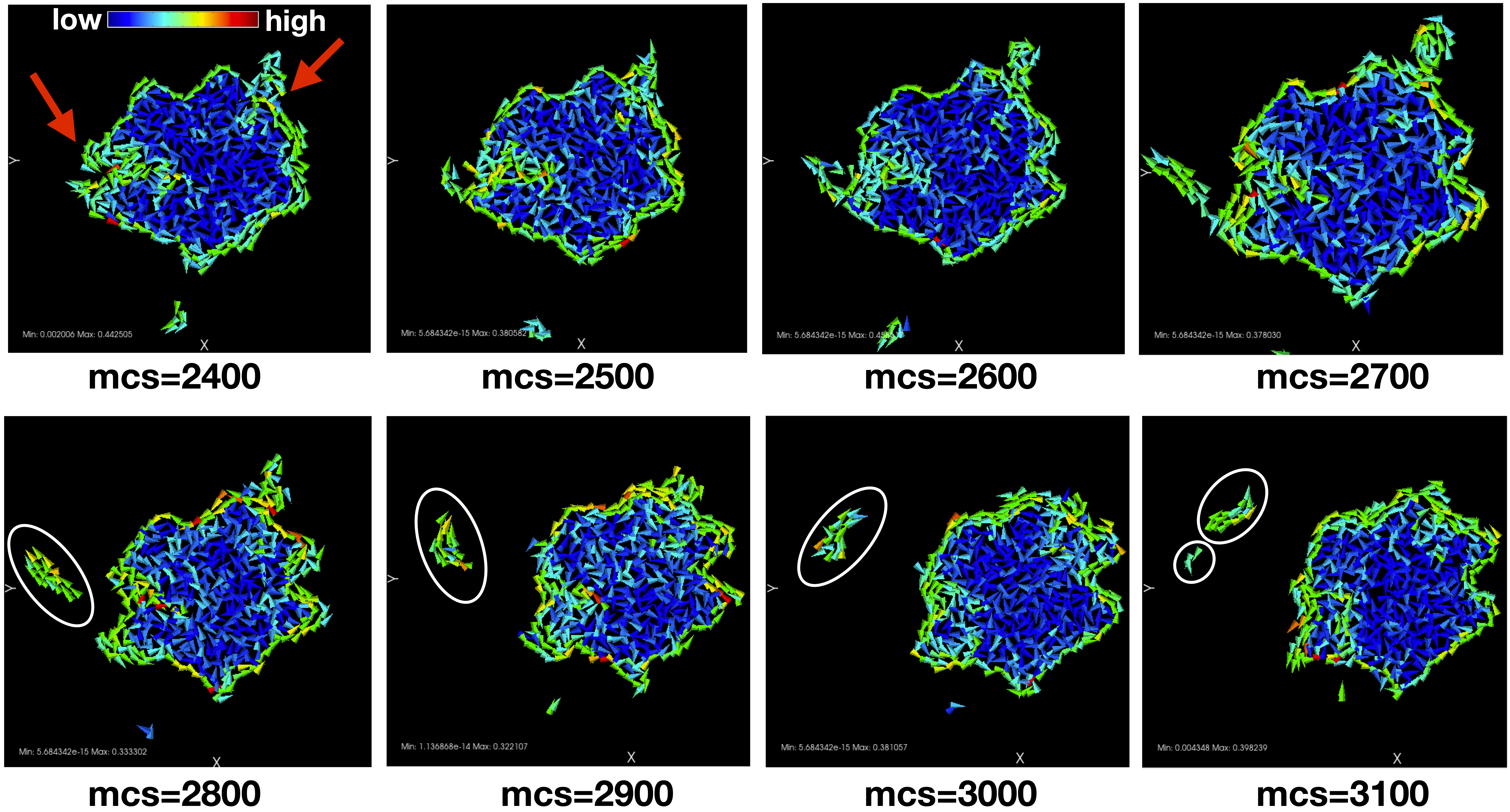

Supplement: S2 Fig — (TIFF) [file pcbi.1009011.s005.tiff]

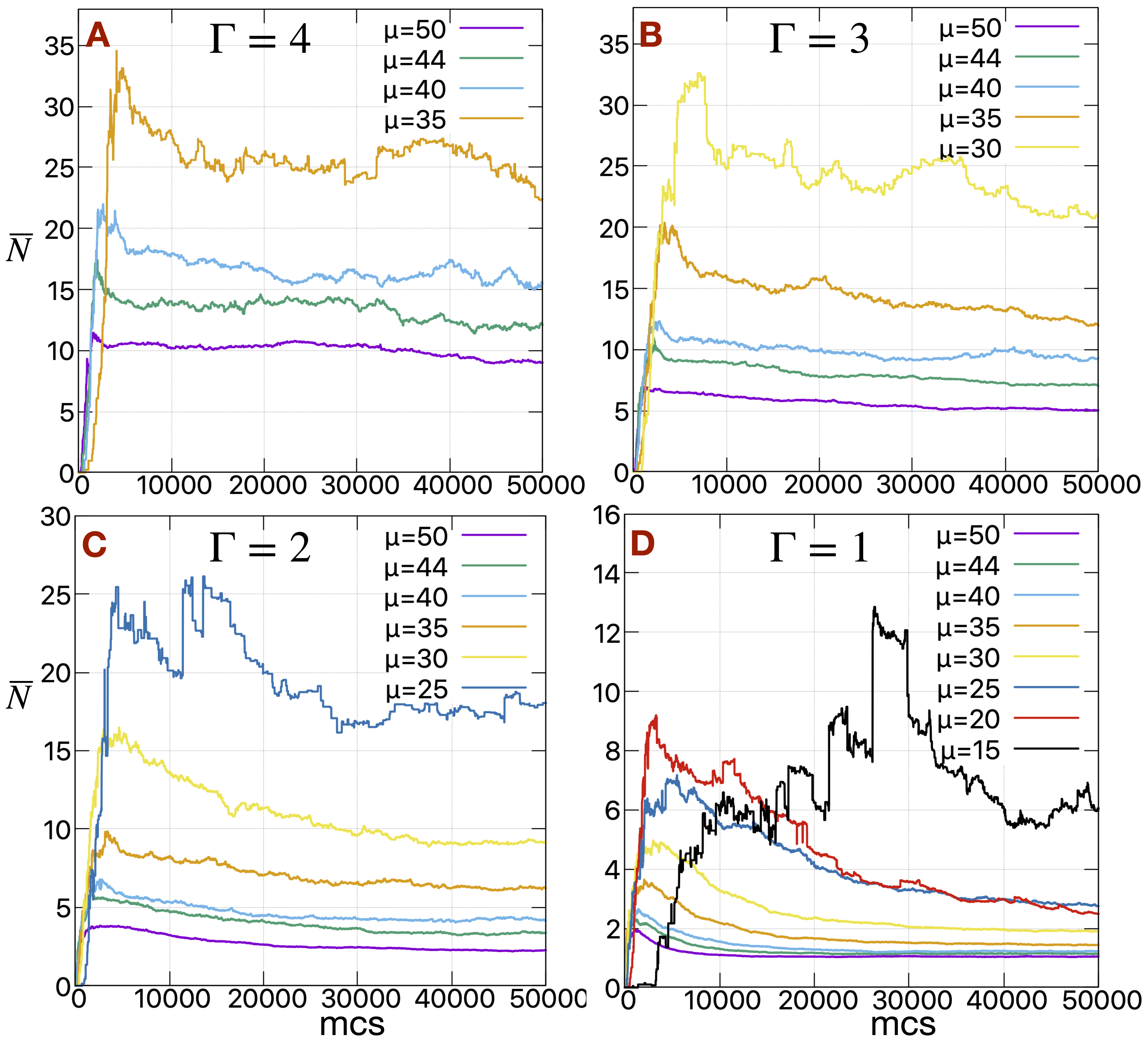

Supplement: S3 Fig — The time series of mean cluster size (N¯) of H cells for different values of μ at (A) Γ = 4, (B) Γ = 3, (C) Γ = 2 and (D) Γ = 1. (TIFF) [file pcbi.1009011.s006.tiff]

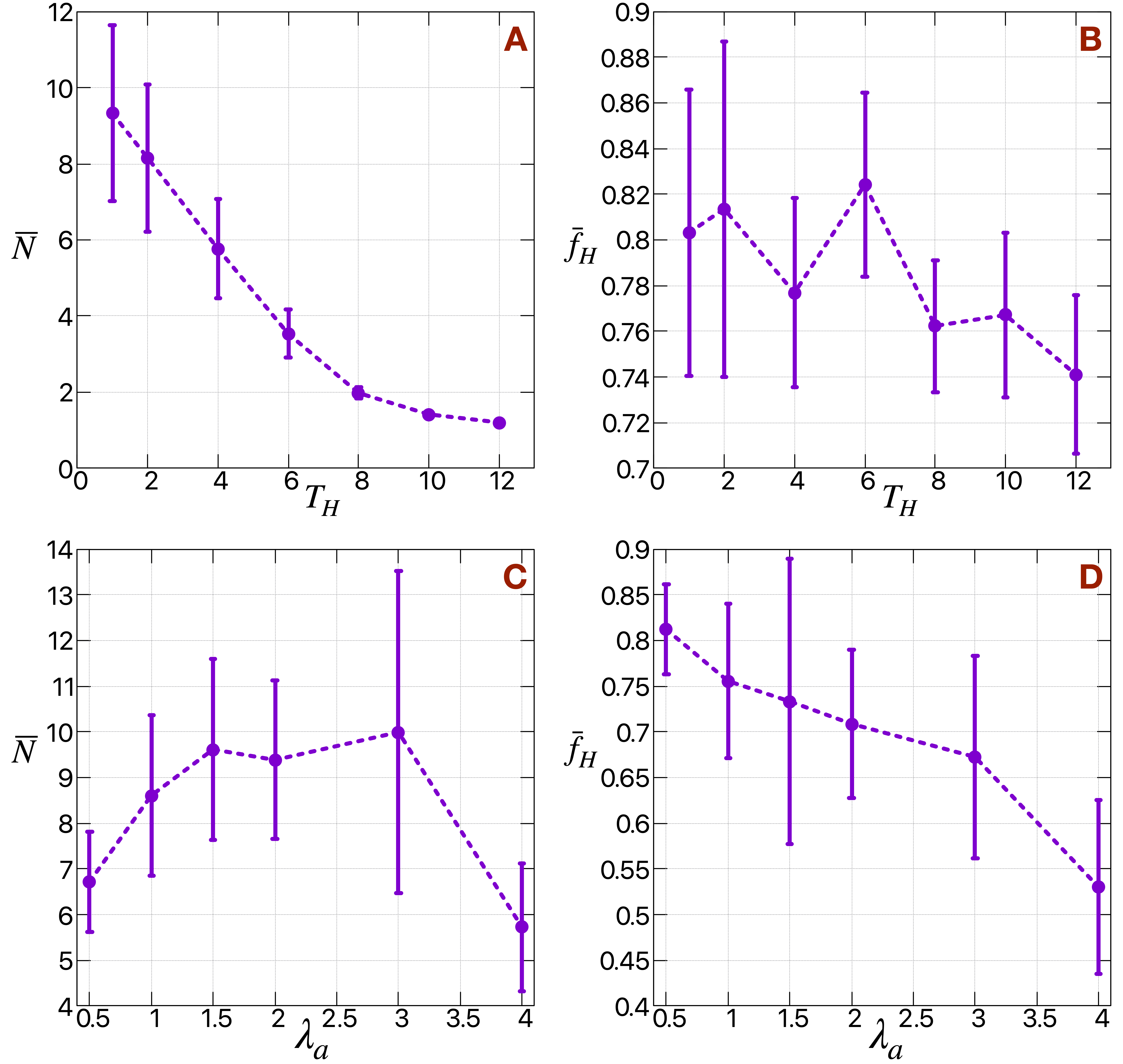

Supplement: S4 Fig — The (A) mean cluster size (N¯) of H cells and (B) fraction of H cells (f¯H) released in the medium as a function of TH at λa = 1. The (C) mean cluster size (N¯) of H cells and (D) fraction of H cells (f¯H) released in the medium as a function of bulk modulus of H cells λa at TH = 1. In all cases μ(H) = 40 and γHm = Γ = 3. We use standard deviations of the data as error bars. (TIFF) [file pcbi.1009011.s007.tiff]

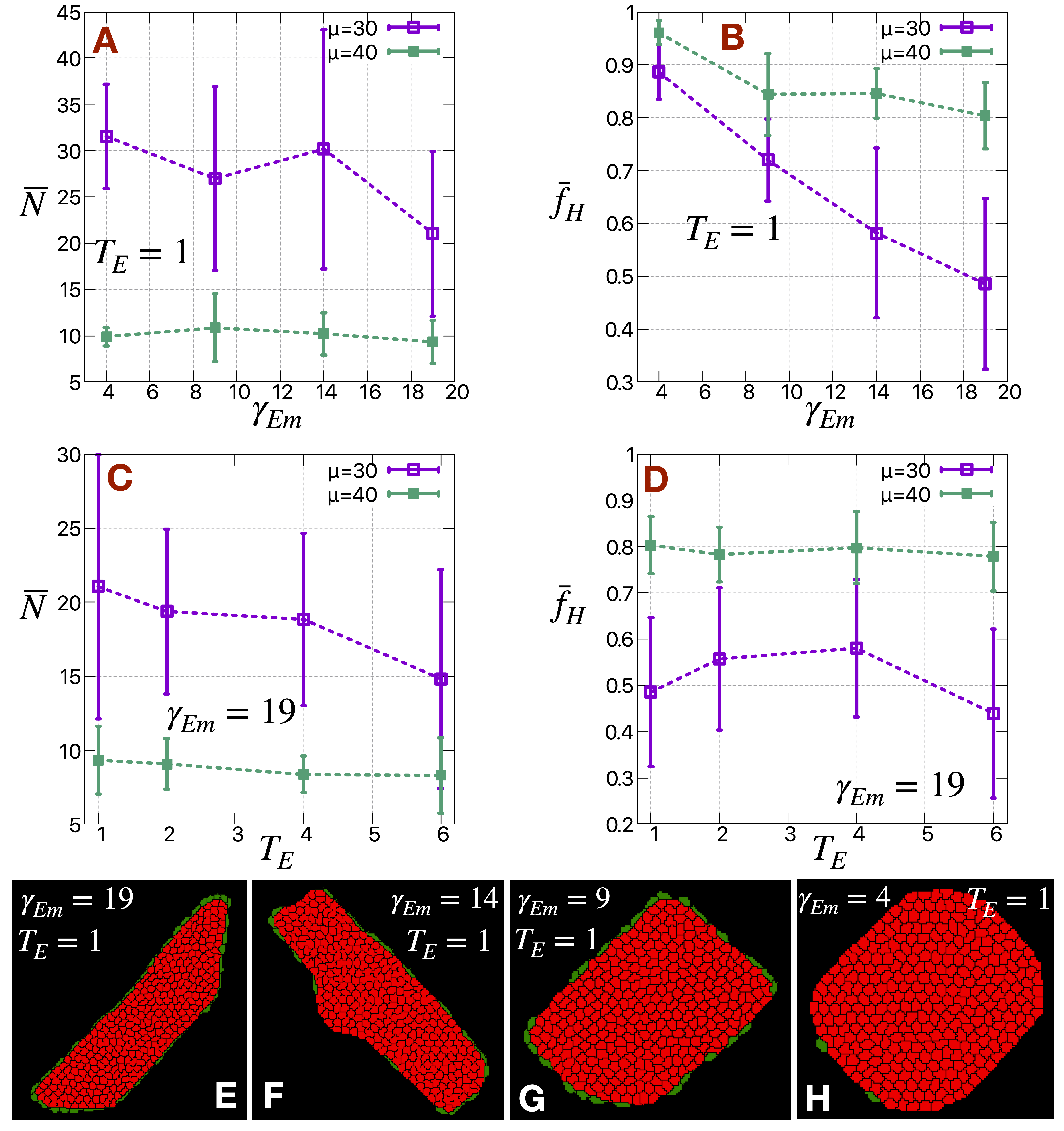

Supplement: S5 Fig — The (A) mean cluster size (N¯) of H cells and (B) fraction of H cells (f¯H) released in the medium as a function of γEm at TE = 1. The (C) mean cluster size (N¯) of H cells and (D) fraction of H cells (f¯H) released in the medium as a function of TE at γEm = 19. In all cases γHm = Γ = 3, TH = 1 and μ(H) = 30 and 40. Representative snapshots of the primary tumor at final step (mcs = 50000) of simulation after most of the H cells are released for different values of γEm = (E) 19, (F) 14, (G) 9 and (H) 4 at TE = 1. We use standard deviations of the data as error bars. (TIFF) [file pcbi.1009011.s008.tiff]

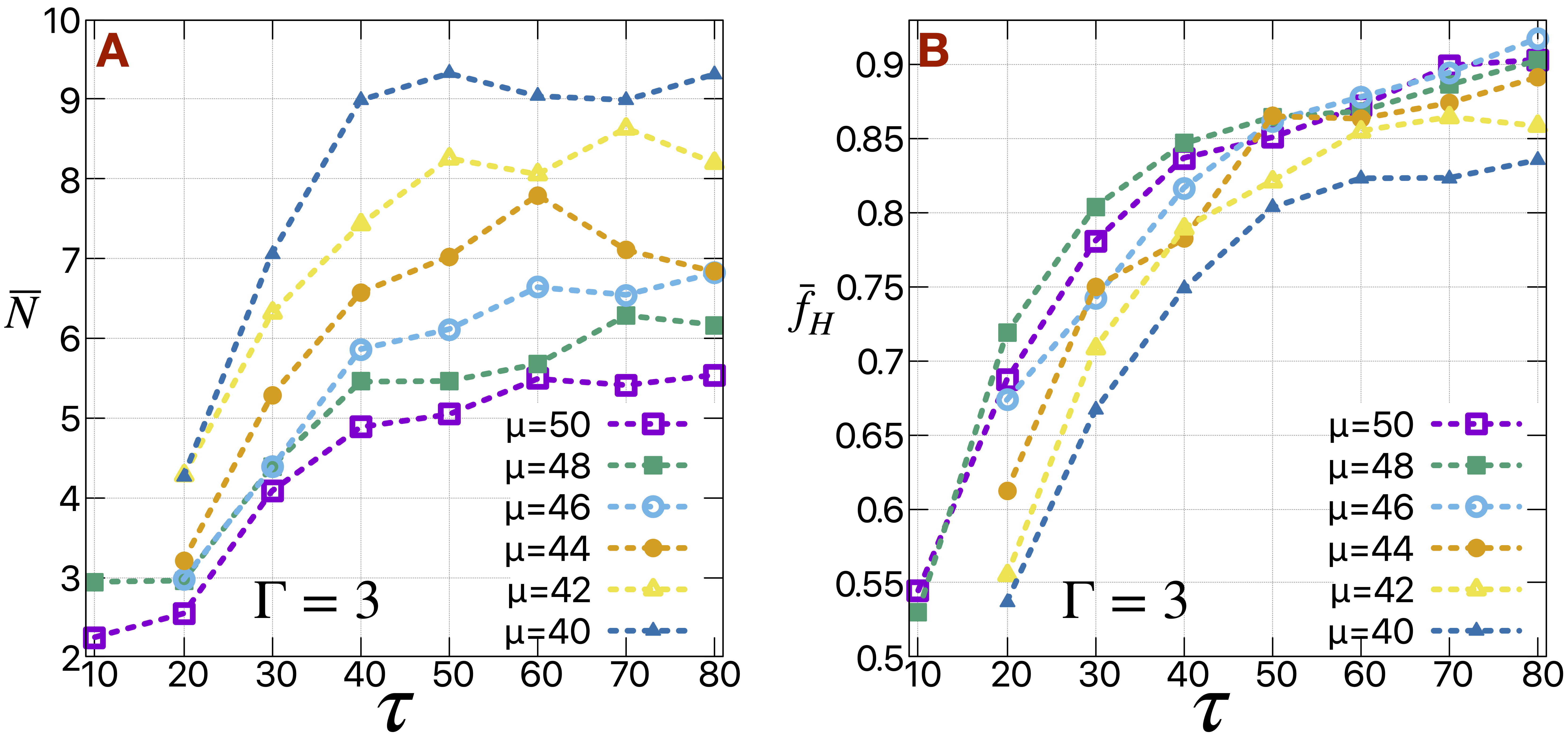

Supplement: S6 Fig — The (A) mean cluster size (N¯) of H cells and (B) fraction of H cells (f¯H) released in the medium as a function of τ for different values of μ at Γ = 3. (TIFF) [file pcbi.1009011.s009.tiff]

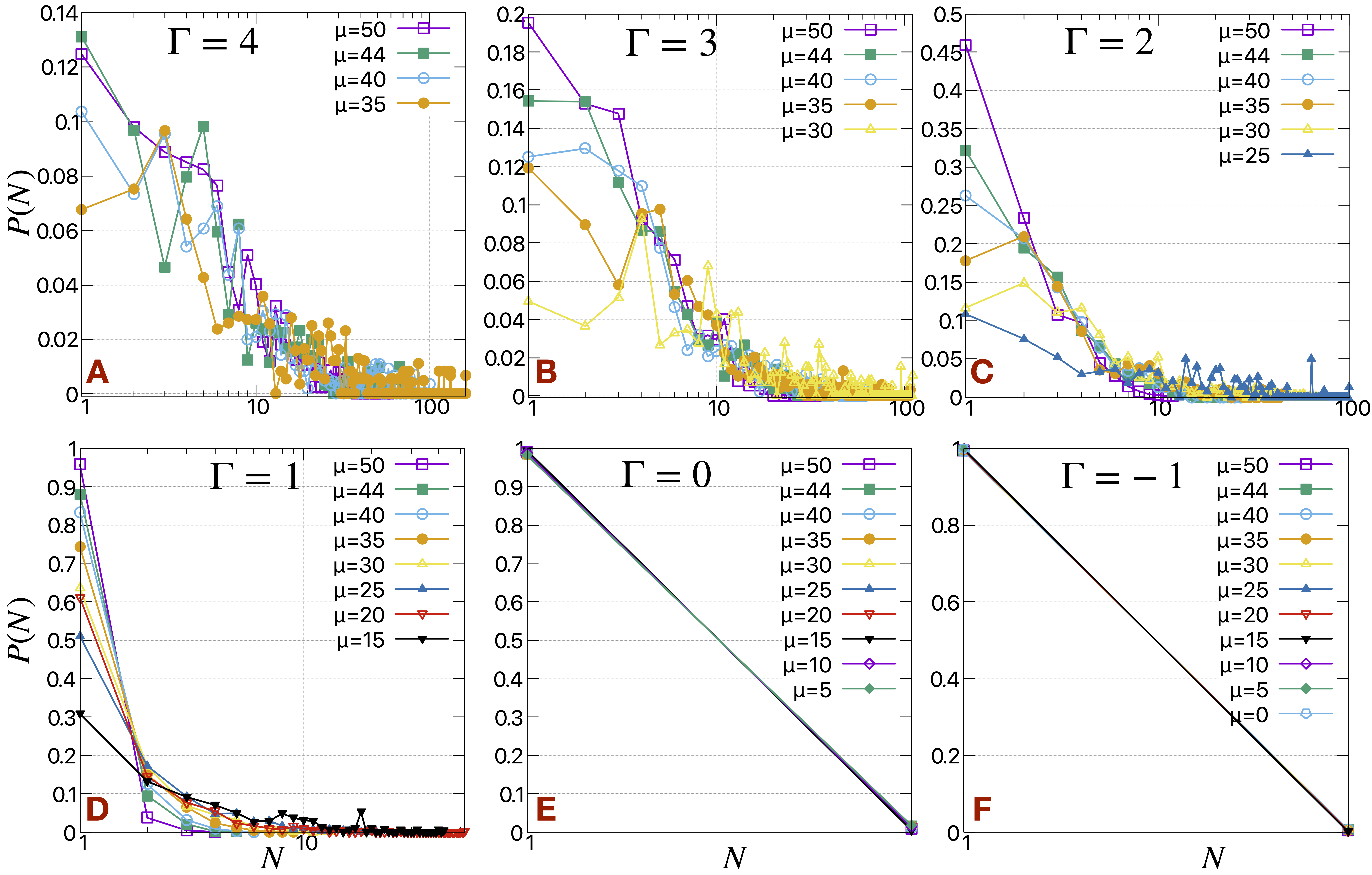

Supplement: S7 Fig — Cluster size distributions (P(N)) of H cells for different values of Γ = (A) 4, (B) 3, (C) 2, (D) 1, (E) 0 and (F) −1. The distributions are normalized such that ∑ P(N) = 1. The N axes are in log scale. (TIFF) [file pcbi.1009011.s010.tiff]

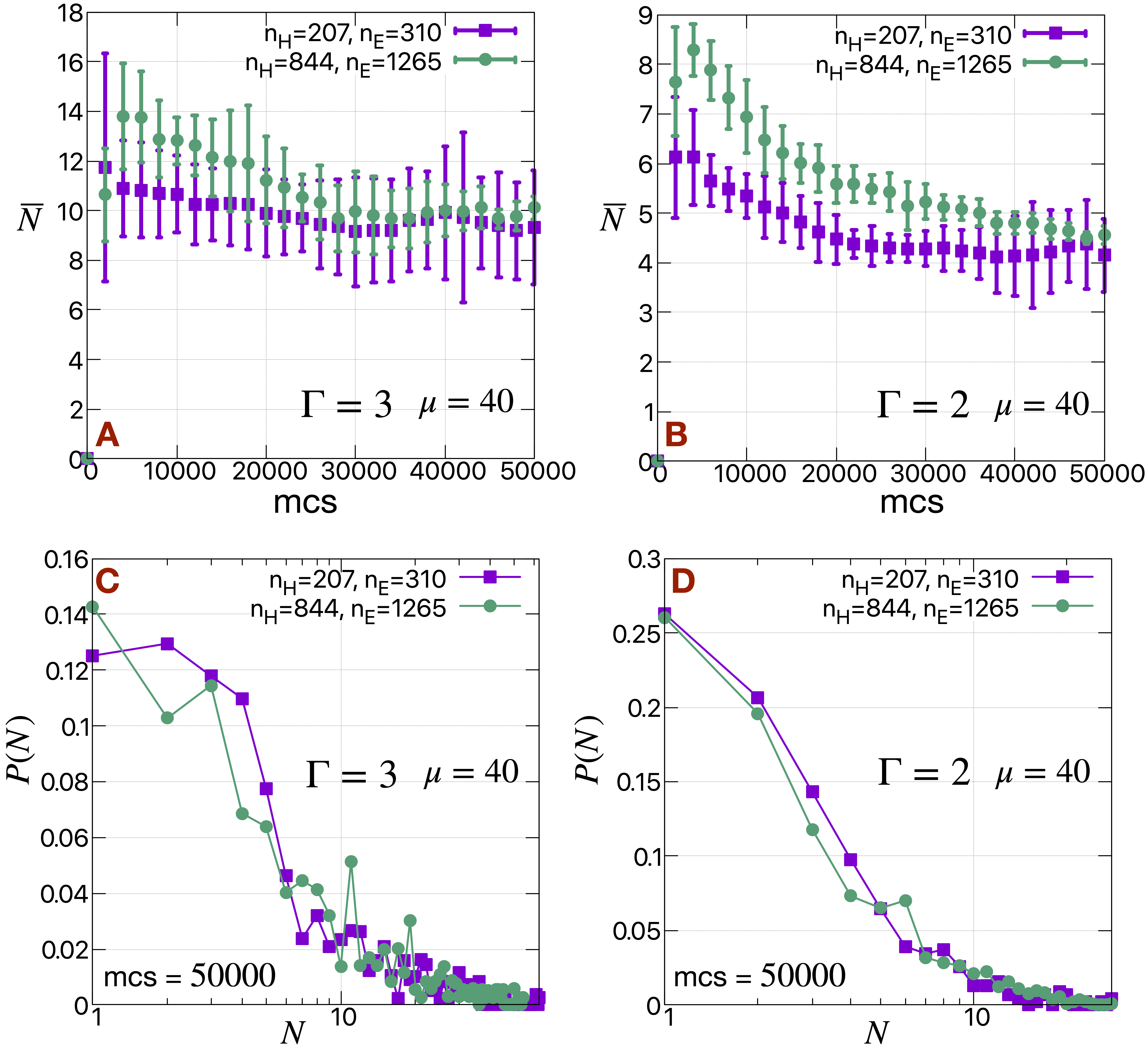

Supplement: S8 Fig — The mean cluster size (N¯) of H cells at (A) μ = 40, Γ = 3 and (A) μ = 40, Γ = 2 for different size of the tumor with total number of cells 517 and 2109 composed of 60% nonmotile E cells and 40% motile H cells. We use standard deviations of the data as error bars. (C) and (D) The corresponding cluster size distributions (P(N)) of H cells. The distributions are normalized such that ∑ P(N) = 1. The N axes are in log scale. (TIFF) [file pcbi.1009011.s011.tiff]

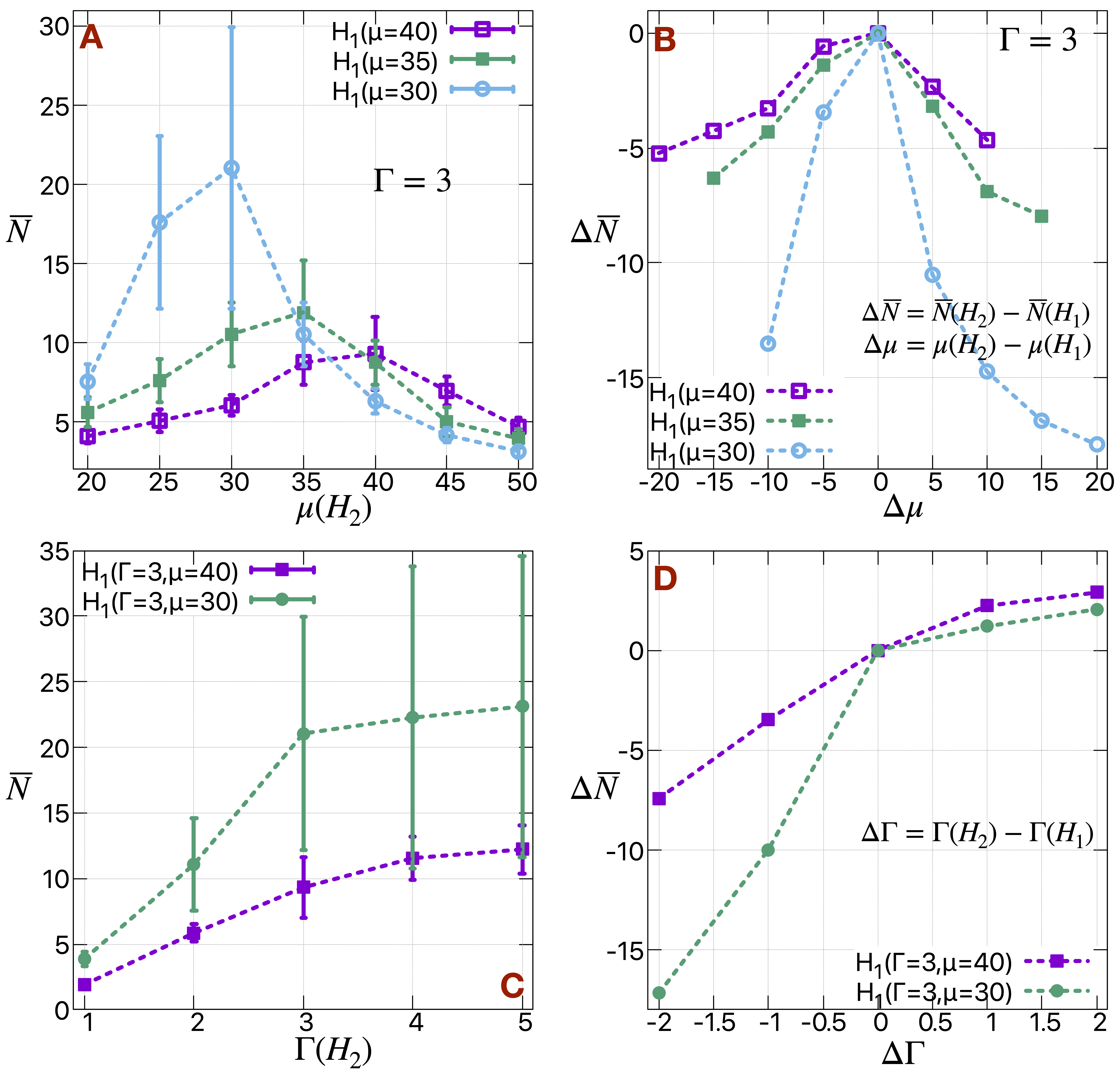

Supplement: S9 Fig — (A) The mean cluster size (N¯) of H cells as a function of μ(H2) in case of two kinds of H cells different in their motile forces at a fixed Γ = 3 for different values of μ(H1). (B) The corresponding difference of N¯ (ΔN¯=N¯(H2)-N¯(H1)) as a function of Δμ = μ(H2) − μ(H1). (C) The mean cluster size (N¯) of H cells as a function of γ(H2) in case of two kinds of H cells different in their cell-medium surface tensions (γ) for μ(H1) = μ(H2) = 30 and 40 at Γ(H1) = 3. (D) The corresponding difference of N¯ (ΔN¯=N¯(H2)-N¯(H1)) as a function of ΔΓ = Γ(H2) − Γ(H1). We use standard deviations of the data as error bars. (TIFF) [file pcbi.1009011.s012.tiff]

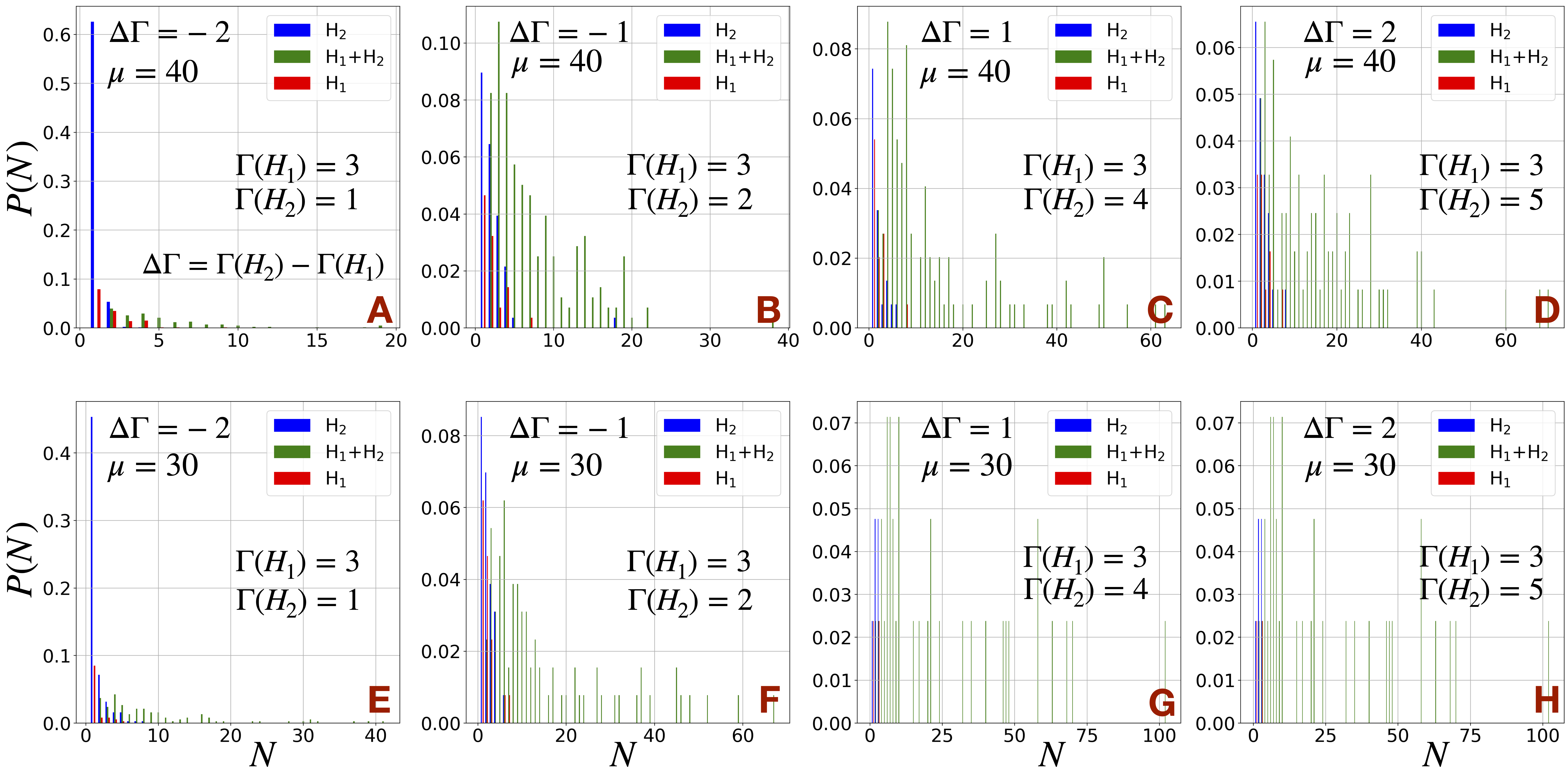

Supplement: S10 Fig — The cluster size distributions (P(N)) of H cells in case of two kinds of H cells different in their cell-medium surface tensions (Γ) at a fixed (A-D) μ = 40 and (E-H) μ = 30 for different values of ΔΓ = Γ(H2) − Γ(H1) by changing Γ(H2) keeping fixed Γ(H1) = 3. At each N, P(N) is splitted into three bars. Red bars represent the clusters consist of only H1 cells, blue bars represent the clusters consist of only H2 cells and green bars represent the heterogeneous clusters consist of H1 and H2 cells. The distributions are normalized such that ∑ P(N) = 1. (TIFF) [file pcbi.1009011.s013.tiff]

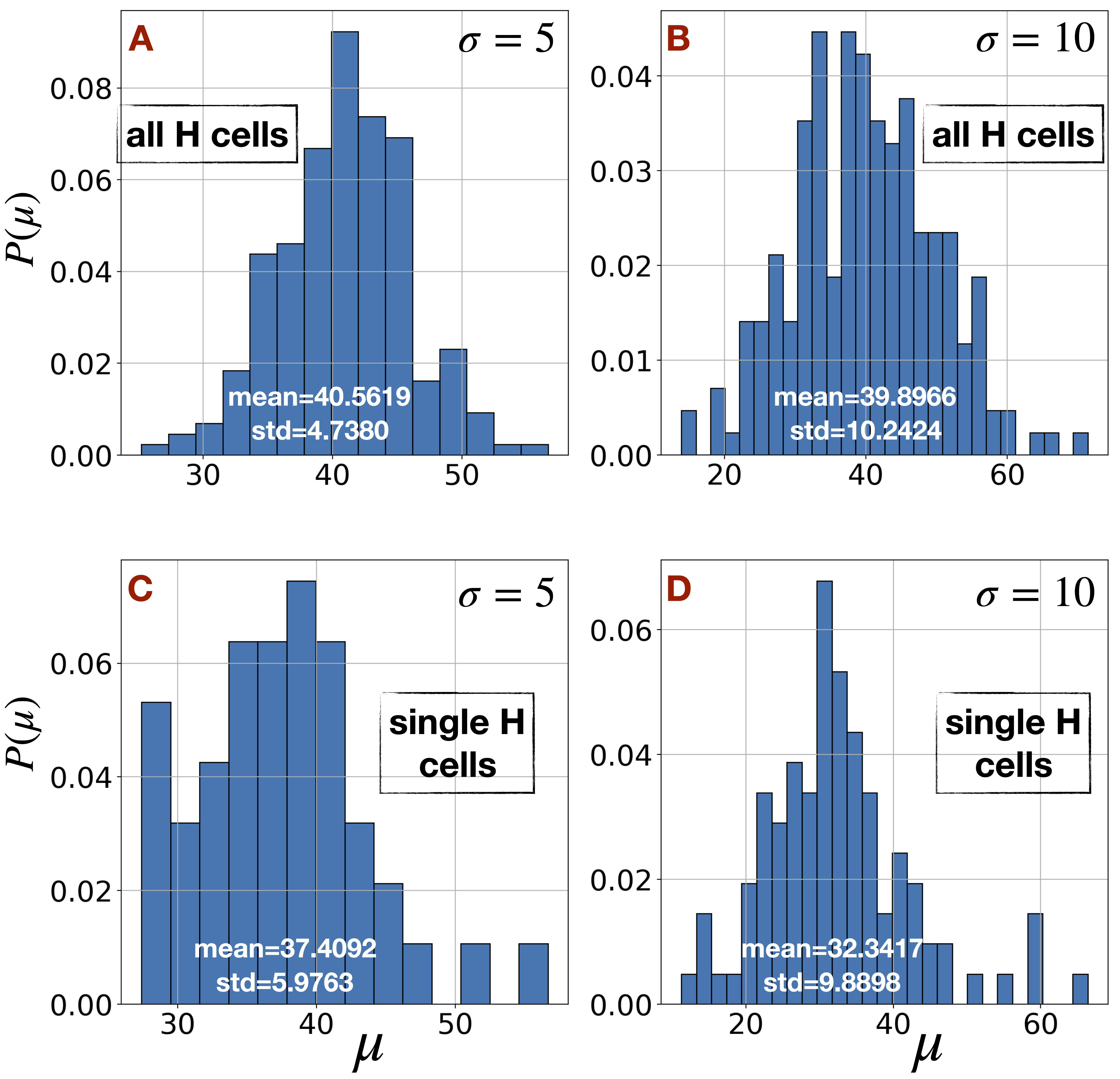

Supplement: S11 Fig — The distribution of motile forces (μ) of all the H cells for (A) σ = 5 and (B) σ = 10, when motile forces of all the H cells are drawn from a normal distribution of mean 40 and standard deviation σ. (C-D) The similar distributions of μ for single H cells (N = 1) in the medium. (TIFF) [file pcbi.1009011.s014.tiff]

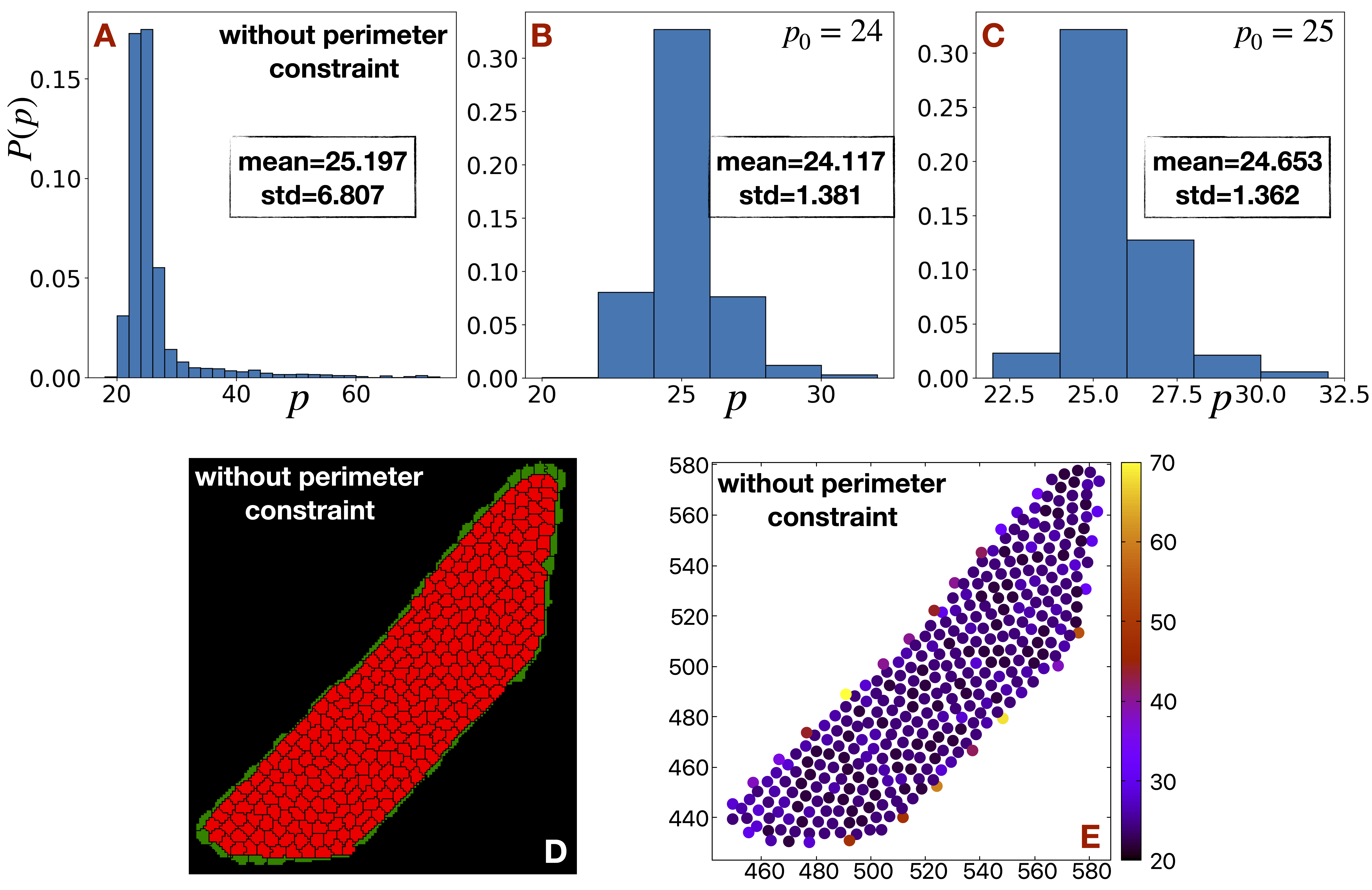

Supplement: S12 Fig — The distribution of perimeters of all the H cells in case of (A) the Hamiltonian without perimeter constraint, the Hamiltonian with perimeter constraint and targeted perimeter (B) p0 = 24, (C) p0 = 25. (D) Representative snapshot of the primary tumor at final step (mcs = 50000) of simulation after most of the H cells are released. (E) The heat map of perimeters of all the cells in the primary tumor at the final step. (TIFF) [file pcbi.1009011.s015.tiff]

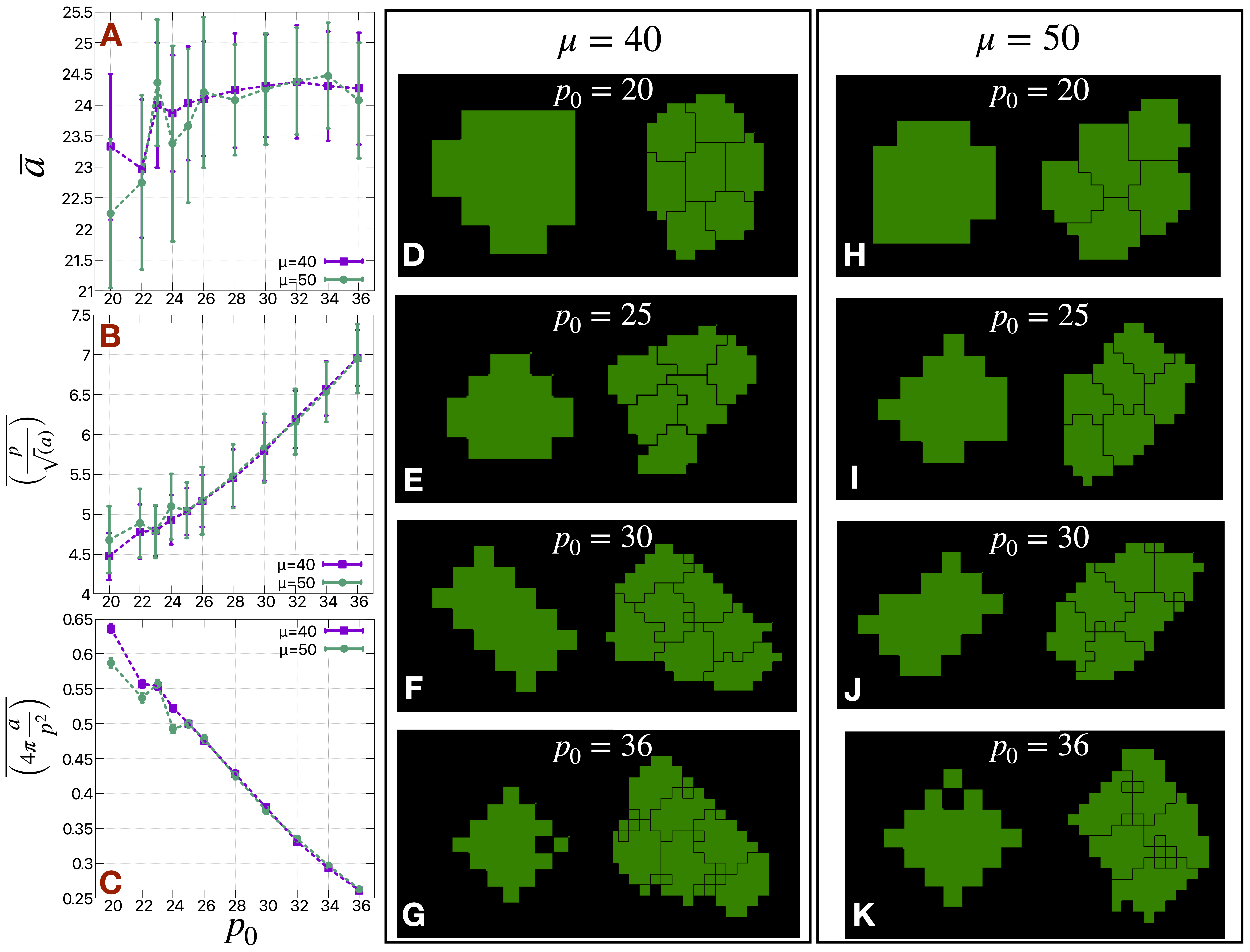

Supplement: S13 Fig — The mean (A) area (a¯), (B) shape index (p(a))¯ and (C) circularity (4πap2)¯ of all the H cells as a function of targeted perimeter (p0). We use standard deviations of the data as error bars. The representative snapshots of the single H cells and small H cell clusters at different values of p0 in case of (D-G) μ = 40 and (H-K) μ = 50. (TIFF) [file pcbi.1009011.s016.tiff]
