# Supplementary material for: Cluster size distribution of cells disseminating from a primary tumor
Source: PLoS Comput Biol. 2021 Nov 10;17(11):e1009011. doi: 10.1371/journal.pcbi.1009011 (PMC8608333; doi:10.1371/journal.pcbi.1009011)
Supplement: Attachment — Submitted filename: Report PCOMPBIOL-D-21-00759 2021.pdf [file pcbi.1009011.s017.pdf]

Report of PCOMPBIOL-D-21-00759
Cluster Size Distribution of Cells Disseminating from a Primary Tumor

The authors numerically investigate the statistics of migrating clusters detaching from primary tumor composed of both motile and non-motile cells. The degree of mobility of the cells is modeled by two factors, the active motile forces of the cells (μ) and cell-medium surface tension (Γ). Simulations are performed using the Cellular Potts Model, a standard model of multicellular systems.

The study is well-done; the background and interpretation are relatively well-explained. The results presented here are new and certainly worth publishing. Further, it is a good fit to the journal.

Some clarifications are needed prior to publication, as described below.

- Some parameters are not defined or their values are not given. In particular, the authors seem to use two different simulation temperatures, named T_E and T_H. This is an unusual way to run cellular Potts simulations. Authors should provide here more explanations on how simulations are run, and why and how these two temperatures are used in the algorithm. Are the two temperatures used for each cell type, respectively (then, explain why) ? But in that case which temperature is used for a lattice site at the boundary between two cells of different type ?

Similarly, the authors should indicate which neighbourhood they used in the simulation lattice to calculate the energy. This info is necessary to estimate the contact energy.

- Cellular Potts model is known to induce fragmentation of cells (see e.g; Durand & Guesnet, Comp. Phys. Comm. 208 (2016)). These fragments can alter the kinetics of cells (see dx.doi.org/10.1371/journal.pcbi.1008576 to appear). The authors should check that the temperature simulations they used are in the range in which fragmentation events are rare.

- During metastasis, tumoral cells are known to divide a lot. Surprisingly, cell division is not mentioned in the manuscript. The authors should comment on this point and whether cell division would affect the results.

- The number of cells used in simulations (517) is very small. Although the results are probably qualitatively corrects, the statistics on migrating clusters must be affected by finite size effects. In particular, power-laws reported in the manuscript should be presented with caution. Running simulations of larger systems would be helpful to confirm these results.

Minor comments :

p. 15, line #419: “Although…,Nevertheless…”: the sentence needs to be rephrased.
