# Supplementary material for: Cluster size distribution of cells disseminating from a primary tumor
Source: PLoS Comput Biol. 2021 Nov 10;17(11):e1009011. doi: 10.1371/journal.pcbi.1009011 (PMC8608333; doi:10.1371/journal.pcbi.1009011)
Supplement: Attachment — Submitted filename: response.pdf [file pcbi.1009011.s018.pdf]

Roeland M.H. Merks, Ph.D
Associate Editor
PLOS Computational Biology

Douglas Lauffenburger
Deputy Editor
PLOS Computational Biology

Revision for (PCOMPBIOL-D-21-00759) - [EMID:a7f83adb82c1c19f]
"Cluster Size Distribution of Cells Disseminating from a Primary Tumor" by Mrinmoy Mukherjee and Herbert Levine

Dear Editors,

We appreciate that the manuscript was found to be of potential interest for the readership of the journal. We thank the referees and the editors for their valuable comments aimed at helping us to improve the manuscript. Below, we address the specific comments of the referees. We have revised the manuscript accordingly and uploaded the new version in the portal.

Mrinmoy Mukherjee and Herbert Levine

****

Response to the Comments of the Referee.

(Legend: in black the text from the referees, in blue our reply)

I. RESPONSE TO THE REVIEWER: 1

In this study the authors carried out numerical simulations of cellular Potts model to investigate cluster size of disseminating cells from primary tumor. It was confirmed that the cluster size increases for larger adhesion while decreases for larger motility, for which scaling relation were determined. Mixture of different cell types were also investigated, where combination of different cell types can facilitate larger cluster size.

Overall, the author has shown many results in various simulation settings, but the analysis is rather descriptive for each setting and the results are not very surprising. I'm not sure if the results are general and / or insightful enough to understand the actual process of metastasis.

1. Some figures are too small and messy, due to which I'm afraid that I misunderstand the results, e.g., Figs. 3C,D, 4F, 5, 6.

Author reply: We have tried to make the figures more clear and comprehendible. We have modified the Fig. 3C,D to make it easier to read. Next, we have changed the Fig. 4 to present in more detail the comparison with the experimental results. In case of Fig. 5, we have represented the cells by their center of mass. This was done because we want to show the full simulation box in order to capture entire releasing and breaking events of clusters. For more resolved observations, please see the video S1 and S2 as these figures are snapshots from the videos. Finally, we have modified the captions of the figures whenever necessary (see the text in red in the Figure captions).

2. Comparison with experimental data are scattered in the main text and are not sufficiently addressed. For example, I'm not sure whether experimental data shown in Fig. 3f are similar to numerical results. The authors should make a more detailed discussion of the interpretation of the obtained results in the light of experimental observation. This will help the readers understand the motivation and purpose of this study.

Author reply: In the revised manuscript, we have modified Fig. 4 to directly compare cluster size distributions from our simulation with CTC cluster distributions taken from different experimental papers (see the text in red on

Page 9–10). We see rather good agreement, especially regarding the trends in the distributions, with the experimental results. We have also included a new paragraph discussing future possibilities for new experiments to compare with our simulation predictions (see the text in red on Page 16)

3. What is the biological meaning of the parameter tau? Is it possible to experimentally disturb it?

Author reply: There is an extensive literature on modeling actively moving cells as persistent random walkers. This approach assumes that cells maintain a fixed direction in which they exert active traction for a time τ; biophysically, this represents a correlation time for the state of cytoskeletal organization, after which the directional polarity spontaneously shifts. We have now included two references to this idea; Gorelik, Roman, and Alexis Gautreau. "Quantitative and unbiased analysis of directional persistence in cell migration." Nature protocols 9.8 (2014): 1931-1943. and Li, Liang, Edward C. Cox, and Henrik Flyvbjerg. "'Dicty dynamics': Dictyostelium motility as persistent random motion." Physical biology 8.4 (2011): 046006. Experimentally, this time scale could be affected by the nature of the extracellular material though which the cell moves, as shown for example in Kim, Jihan, et al. "The mechanics and dynamics of cancer cells sensing noisy 3D contact guidance." Proceedings of the National Academy of Sciences 118.10 (2021), as well as by altering the organization of the cytoskeleton (by using cells with a different level of EMT). We have added some remarks to this effect in the (new) discussion of possible future experiments (see Page 16).

4. The author numerically determined powers of scaling behaviors of N and f on mu and Gamma. Are these values are universal, in other words, simulation with other model schemes can also reproduce the same results? Is it possible to theoretically derive these powers?

Author reply: We believe this scaling relation is should be relatively general, and hence could be generated by other classes of models with the necessary components (adhesion and motility) described in CPM. Unfortunately, we do not at present have a theoretical derivation or a proof of the above conjecture; hopefully, publication of this simulation study will study will spur further efforts (including ours) along those lines.

5. How μ_{min} scales with Gamma? Using μ–μ_{min} instead of μ seems more natural choice (I might be wrong).

Author reply: We didn't try to scale with μ–μ_{min} here because the boundary of μ_{min} was not sharply defined given our resolution of $\Delta\mu = 5$ and given the fact that the error in mean cluster size increases as we approach μ_{min}. This could presumably be improved by choosing a larger number of cells in the tumor and small value of $\Delta\mu$ near the boundary of μ_{min}. These changes necessitate many more simulations, which are beyond the scope of this study. Also, when comparing with the experimental cluster size distributions, μ lies in [30,50] and hence we can roughly ignore the μ_{min} issue by choosing a certain range of $\mu > 30$. μ_{min} will only go below 30 only for $\Gamma > 3$, which is not in the range that compares favorably with any of the experimental data.

6. Fig. 7A and L313. The authors claim "N remains almost constant up to sigma = 2, ..", but it is unclear this statement is valid from Figs 7A and B.

Author reply: The probability of getting smaller clusters ($N < 3$) are almost similar for $\sigma < 2$ (see the distributions below the black dotted lines in Fig. 7B). The variation in the mean (Fig. 7A) for $\sigma < 2$ is mainly due to variation in probability of getting larger clusters ($N > 3$). Also, see the sharp fall of mean cluster size for $\sigma > 2$.

7. Fig. 7D and E: adding error bars is preferable.

Author reply: The error bars (standard deviation) is actually plotted in Fig. 7E. And the mean values are plotted in Fig. 7D.

8. The section "Cell shape driven cluster size distribution" seems interesting. Are the results related with jamming transition reported in Bi, et al. Nat. Phys. (2015)?

Author reply: Actually we have discussed our findings with Max Bi and we do not think that they are directly related to jamming transition. As we mentioned in the text, we observe that when the cells can not reach their targeted perimeter (negative cortical tension, cells behave more liquid like), the clusters break easily and on the other hand, at very small value of targeted perimeter cells become too rigid to release from the primary tumor. This appears to us to be distinct from the unjamming due to T1 transitions in an extended epithelial layer.

9. Typos: L226 "fall rapidly fall as N ..", L420 "Nevertheless" N is capital.

Author reply: We have corrected these typos.

II. RESPONSE TO THE REVIEWER: 2

The authors numerically investigate the statistics of migrating clusters detaching from primary tumor composed of both motile and non-motile cells. The degree of mobility of the cells is modeled by two factors, the active motile forces of the cells (μ) and cell-medium surface tension (Γ). Simulations are performed using the Cellular Potts Model, a standard model of multicellular systems.

The study is well-done; the background and interpretation are relatively well-explained. The results presented here are new and certainly worth publishing. Further, it is a good fit to the journal.

Some clarifications are needed prior to publication, as described below.

- Some parameters are not defined or their values are not given. In particular, the authors seem to use two different simulation temperatures, named TE and TH. This is an unusual way to run cellular Potts simulations. Authors should provide here more explanations on how simulations are run, and why and how these two temperatures are used in the algorithm. Are the two temperatures used for each cell type, respectively (then, explain why) ? But in that case which temperature is used for a lattice site at the boundary between two cells of different type ?

Author reply: We agree that this point needs some clarification,. We only use different T for E and H cells in case of diffusive motility (Fig. 1). In particular, we use higher T for H cells in absence of active motility, to make the H cells more motile as compared to E cells; in general the Epithelial (E) cells are very rigid, do not move very much. At the boundary between two cells of different type, the T used as $min(T_E, T_H)$. We have added a statement to the text to make these points more clear (see the text in red on Page 3). For the simulations including active motility of H cells, we use same T.

Similarly, the authors should indicate which neighborhood they used in the simulation lattice to calculate the energy. This info is necessary to estimate the contact energy.

Author reply: We simulate the system in a 2D square lattice, including nearest-neighbor and next-nearest neighbor terms. We have clarified this point in the revised manuscript (see the text in red on Page 3).

- Cellular Potts model is known to induce fragmentation of cells (see e.g; Durand & Guesnet, Comp. Phys. Comm. 208 (2016)). These fragments can alter the kinetics of cells (see dx.doi.org/10.1371/journal.pcbi.1008576 to appear). The authors should check that the temperature simulations they used are in the range in which fragmentation events are rare.

Author reply: Indeed, at very high value of T, μ and p_0, the cells can become fragmented. We work in a region of parameter space, where fragmentation events are very rare. We do observe some fragmentation at $p_0 > 30$ (see Fig. S13 G). We have discussed this in the revised manuscript (see the text in red on Page 3).

- During metastasis, tumoral cells are known to divide a lot. Surprisingly, cell division is not mentioned in the manuscript. The authors should comment on this point and whether cell division would affect the results.

Author reply: Of course, a more complete model (certainly one that might eventually be applied to in vivo situations) should include cell division. Aside from the increased complexity of the model, doing this would require an understanding of the relative growth rates of different cell phenotypes as compared to the rates at which cells leave the tumor. It is true that cells that undergo EMT often reduce their division rate (this is sometimes called "go versus grow") and if so, clusters should not have their size distribution dramatically altered by adding in division. A full (and future) study of this issue would be needed to fully verify the conditions under which this is a safe assumption. In general, however, we agree that not bringing up this issue was an oversight and we have added some material to the discussion section to address this oversight. We have added this discussion in revised manuscript (see

the text in red on Page 16).

- The number of cells used in simulations (517) is very small. Although the results are probably qualitatively corrects, the statistics on migrating clusters must be affected by finite size effects. In particular, power-laws reported in the manuscript should be presented with caution. Running simulations of larger systems would be helpful to confirm these results.

Author reply: As a check of our results, we have carried out simulations for larger system size (number of cells 2109) and observe that the mean cluster size matches with the previous one at mcs=50000 (Fig. S8). We have included this in the revised manuscript (see the text in red on Page 10).

Minor comments :

p. 15, line #419: "Although...,Nevertheless...": the sentence needs to be rephrased.

Author reply: We have corrected this.

III. RESPONSE TO THE REVIEWER: 3

The manuscript presents a detailed study of a cellular Potts model with the aim of chracterizing clusters of metastatic cells in terms of the simulated EMT state of the individual cells comprising the cluster. Understanding how the physical organization of metastatic cells depends on the gene regulated phenotype of the individual cells undergoing EMT is an interesting topic worth investigating. The present computational study could guide further experimental studies. In this respect, I would encourage the authors to discuss in the conclusions possible avenues for experimental investigation of the same problem. In general the manuscript is well written and I would thus suggest publication.

Author reply: In the revised manuscript, we have modified Fig. 4 to compare the cluster size distributions from our simulation with the CTC cluster distributions taken from different experimental papers (see the text in red on Page 9–10). We see rather good agreement, especially the trend in the distributions, with the experimental results. We have also included one paragraph discussing the future possibilities of experiments to compare with our simulation predictions (see the text in red on Page 16).

I have some minor issues that should be fixed before acceptance:

The analysis presented in Fig. 3 should be improved. The author state that the fit in Fig. 3A yields an exponent of 1.98 +- 0.01. Yet, the fit is performed over three points with huge error bars. How is it possible that the error bar on the exponent is so tiny. Was the error on the data included in the estimate of the error bar on the fit (my guess is that the authors reported what the fitting routine gave). Also, it is not clear, here and in other instances what the error bar actually is. It is the SD or the SE on the mean? Please provide the definition of the error bars in all the captions. I have similar considerations for the fit p=0.999 +- 0.001 in Fig. 3c. It is clear that a line fits the data, but I do not believe the tiny error bar. Since the author are fitting multiple curves in a parameter dependent way, the most correct way to proceed is to fit collectively all the curves with a single scaling function with unknown parameters. In this way, all the parameters are fit simultaneously over multiple curves.

Author reply: We use standard deviations of the data as error bars. We modified the caption of all the figures accordingly, whenever necessary (see the text in red in the Figure captions).
For Fig. 3B, we simulate for two extra points ($\Gamma = 2.5, 3.5$). The data are nicely fitted with the power law with the exponent 2.0+-0.2. Previously, the errors in the data were not included properly. We modified the new fitted parameters through out the text. We use the least squares method to fit the data with the errors.
In Fig. 3C, we actually fit all the data collectively using a single scaling function $x = \Gamma^{2.0}/\mu^{2.8}$. We fit the data in two ways, by a straight line $N_0 x$ and by a power law $32470x^p$ as discussed in the manuscript. Now, we get $p = 0.99 \pm 0.01$.
The error is still small. One of the reason can be– in case of least square fitting with errors in the data, the χ^2 (the variance in original data and fit function) is weighted by the errors in the data assuming that the errors are normally distributed. But is presence of large error bars, this method sometime underestimate the errors in the

fitted parameters, especially when the errors in the data are not normally distributed. Large number of data sets can improve the result.
In our case, data fit better in the lower range of x (equivalently the higher range of $\mu = [40, 50]$) and/or lower range of $\Gamma = [1, 3]$. Beyond that, the noise in the data increases. This can be be improved either by large system size (large number of cells or bigger tumor) or by doing more ensemble average.

IV. ADDITIONAL REMARKS

Have the authors made all data and (if applicable) computational code underlying the findings in their manuscript fully available?
Reviewer #1: No: I did not notice code and detailed data list. The results themselves are reproducible by the given explanation.
Reviewer #2: No
Reviewer #3: Yes

Author reply: One sample code is provided in the Github repository `https://github.com/mrinmoy169/Cluster_Size_Distribution.git`. All the data presented in this article can be easily generated by the given parameter set listed in Table S1. We have included this in the revised manuscript (see the text in red on Page 3.)
