# Supplementary material for: Cluster size distribution of cells disseminating from a primary tumor
Source: PLoS Comput Biol. 2021 Nov 10;17(11):e1009011. doi: 10.1371/journal.pcbi.1009011 (PMC8608333; doi:10.1371/journal.pcbi.1009011)
Supplement: Attachment — Submitted filename: response.pdf [file pcbi.1009011.s019.pdf]

Roeland M.H. Merks, Ph.D
Associate Editor
PLOS Computational Biology

Douglas Lauffenburger
Deputy Editor
PLOS Computational Biology

Revision for (PCOMPBIOL-D-21-00759R1) - [EMID:50e3555aebced377]
"Cluster Size Distribution of Cells Disseminating from a Primary Tumor" by Mrinmoy Mukherjee and Herbert Levine

Dear Editors,

We appreciate that the manuscript was found to be of potential interest for the readership of the journal. We thank the referees and the editors for their valuable comments aimed at helping us to improve the manuscript. Below, we address the specific comments of the referees. We have revised the manuscript accordingly and uploaded the new version in the portal.

Mrinmoy Mukherjee and Herbert Levine

****

Response to the Comments of the Referee.

(Legend: in black the text from the referees, in blue our reply)

I. RESPONSE TO THE REVIEWER: 1

The authors answered my comments and questions, and the revised manuscript is much improved. I now agree the manuscript meets publication from the PLOS Compt. Biol.

Author reply: Thank you for finding the article publishable.

II. RESPONSE TO THE REVIEWER: 2

The authors satisfactorily answered my comments, except the one about fragmentation, which has been overlooked. The authors claimed that they work in a region of parameter space where fragmentation events are very rare, as they observe fragmentation at $p_0 > 30$. However, this represents 37.5% of the range they scanned ([20,36]). As this has been shown recently (M. Durand, PLOS Comp. Biol. 17, e1008576, 2021), apparition of fragments can significantly affect the results of CPM simulations. Therefore, the results presented in Fig. 8, S12 and S13 should be presented with proper warnings, at least.

Author reply: We have now addressed the issue of fragmentation of cells in more detail in the revised manuscript (see the text in red on Page 14). We have also added a new reference M. Durand, PLOS Comp. Biol. 17, e1008576, 2021 (see the reference [52] in the revised manuscript).

III. RESPONSE TO THE REVIEWER: 3

The authors modified the paper in order to respond to the remarks of the referees. I am satisfied by the response.

Author reply: Thank you for finding the article good fit for PLOS Computational Biology.
